# False lumen access for trans-septal thoracic endovascular aortic repair in a 10-cm dissecting thoracoabdominal aortic aneurysm

**DOI:** 10.1016/j.jvscit.2021.10.013

**Published:** 2021-11-11

**Authors:** Julia Fayanne Chen, Prasanth Vallabhajosyula, Naiem Nassiri

**Affiliations:** a Division of Vascular Surgery and Endovascular Therapy, Department of Surgery, Yale University School of Medicine, New Haven, Conn; b Division of Cardiac Surgery, Department of Surgery, Yale University School of Medicine, New Haven, Conn

**Keywords:** Aortic dissection, False lumen, TEVAR, Thoracoabdominal aortic aneurysm, Trans-septal

## Abstract

Endovascular treatment of the chronically dissected aorta can be especially challenging due to unending variations in post-dissection configurations. Traditionally, basic principles of thoracic endovascular aortic repair rely on bilateral femoral access and deployment of a stent-graft within the true lumen. In the present report, we describe a case of trans-septal thoracic endovascular aortic repair in a patient with complex chronic residual type B aortic dissection (1,10) with dilation up to 10 cm in the context of a chronically occluded right external iliac artery, and a left iliofemoral system supplied by the false lumen.

Thoracic endovascular aortic aneurysm repair (TEVAR) is now the first-line treatment of descending thoracic aortic aneurysms and dissection.^1^, ^2^, ^3^, ^4^ The basic criteria for TEVAR eligibility include adequate proximal and distal landing zones and adequate iliofemoral access.^5^ Cases of aortic dissection can be especially challenging due to the presence of multiple fenestrations and dual lumens. In the present report, we describe a case of trans-septal TEVAR for a patient with complex chronic residual type B aortic dissection (1,10) with dilation up to 10 cm after previous ascending aortic replacement for acute type A dissection, in addition to chronic complete occlusion of the right external iliac artery and a left iliofemoral system supplied by the false lumen. The patient provided written informed consent for the report of her case details and associated imaging studies.

## Case report

**Fig 1:**
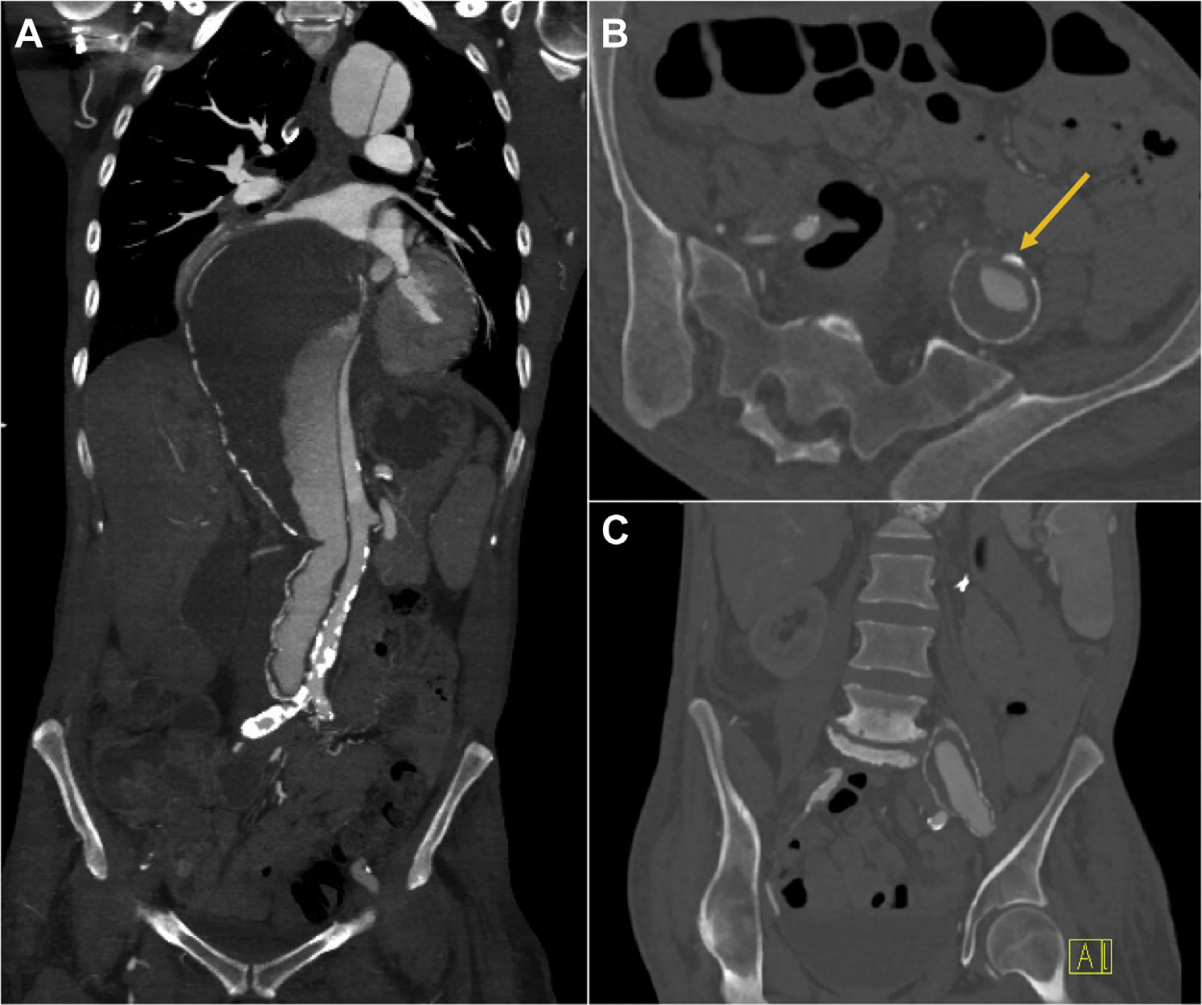
**A,** Chronic residual dissection of the arch and descending and abdominal aorta with aneurysmal dilation up to 10 cm of the distal descending thoracic aorta. **B, C,** Occluded right external iliac artery, patent right internal artery, with the left mid-to-distal common and external iliac arteries supplied entirely by the false lumen. *Arrow* points to a spot of calcium representative of the only remnant of the true lumen in the distal left common iliac artery **(B)**. Contrast enhancement can be visualized in the false lumen.

Our patient is a 49-year-old female smoker with history of hypertension, coronary artery disease, coronary artery bypass grafting, renal cell carcinoma, nephrectomy, end-stage renal disease requiring hemodialysis, and stroke with residual left lower extremity paralysis. She had previously undergone ascending aortic replacement for acute type A dissection 14 years earlier. On presentation, computed tomography angiography (CTA) demonstrated residual dissection of the arch and entire thoracoabdominal aorta with aneurysmal degeneration measuring 10 cm in maximum diameter (Fig 1, *A*). CTA also revealed complete occlusion of the right external iliac artery and a left iliofemoral system supplied entirely by the false lumen (Fig 1, *B* and *C*).

In conjunction with the cardiac team, a multistage approach was devised. This consisted of (1) total arch replacement with great vessel debranching; (2) left carotid–subclavian bypass; and (3) TEVAR (0,5) with coil embolization of the left subclavian artery. Additional interventions to address the perivisceral and infrarenal portions of the aorta would be contingent on the result of aortic remodeling after these initial interventions. Noted challenges included a third-time redo sternotomy and the lack of normal femoral access. After extensive discussion with the patient, the cardiac surgery team proceeded with the first stage of the operative plan. After full recovery, the patient was brought back 5 months later for the next two stages. Left carotid–subclavian bypass was performed without complications, and 2 days later, the patient returned for elective TEVAR.

**Fig 2:**
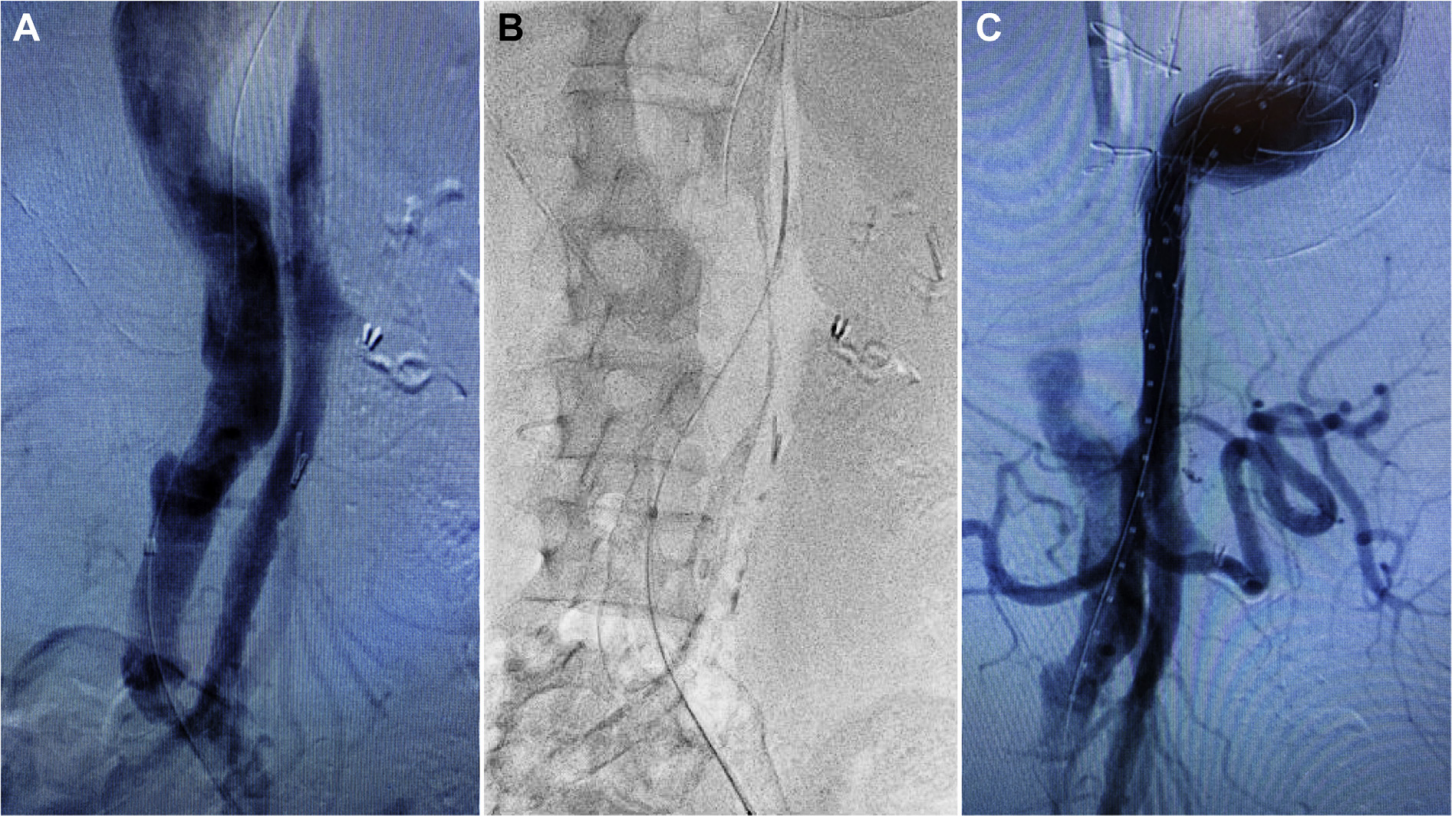
**A,** Angiogram demonstrating numerous large fenestrations connecting the true and false lumens. **B,** Under roadmap guidance, the proximal-most fenestration was crossed using an angled Glidewire and a Berenstein catheter, allowing access into the true lumen. **C,** Catheter placement in the true lumen was confirmed via angiography.

**Fig 3:**
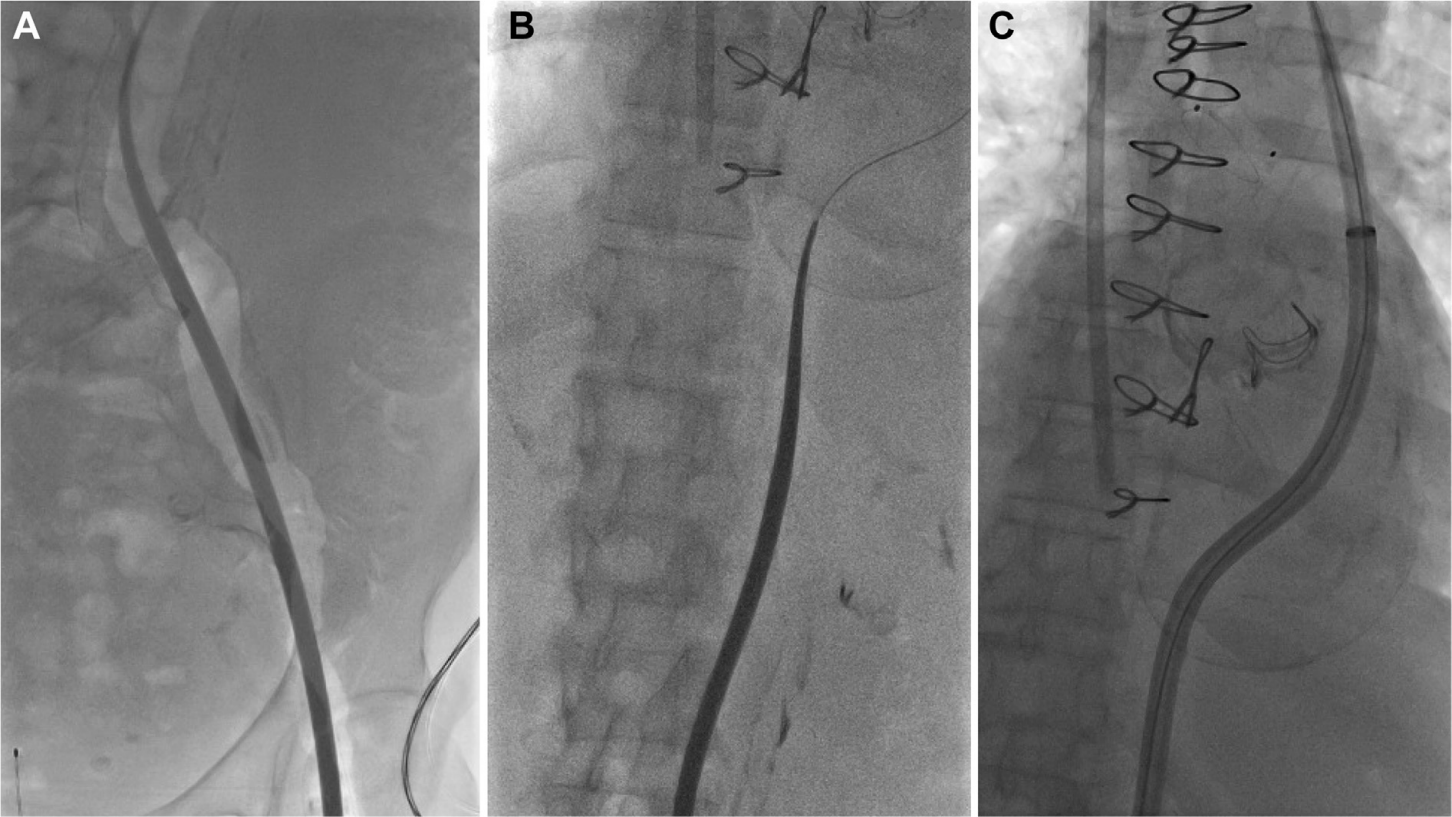
Serial dilation of the fenestration up to 18F was performed. Dilators were inserted via the left femoral access site **(A)** and passed across the fenestration into the true lumen **(B)**. **C,** A 20F Gore DrySeal sheath was then inserted across the fenestration with the tip positioned in the true lumen.

**Fig 4:**
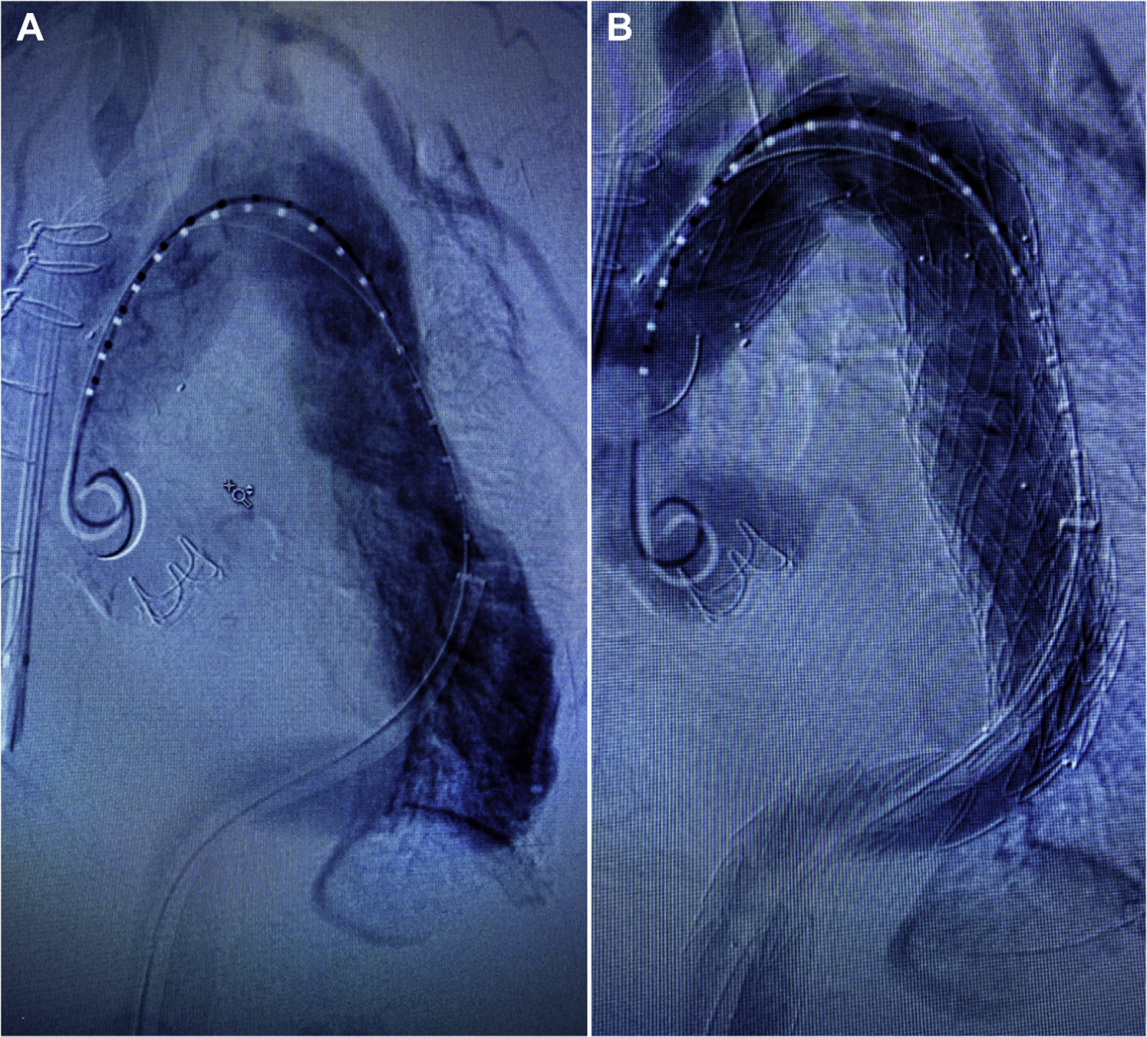
**A,** Ascending and arch angiogram demonstrated the dilated descending thoracic aorta and significant false lumen filling. **B,** Completion angiogram demonstrated immediate augmentation of the true lumen with patent cervical and visceral vessels and no antegrade flow into the false lumen.

The patient was brought to the hybrid operating room suite, a lumbar drain was placed, and continuous neuromonitoring via somatosensory evoked potentials (SSEP) and electroencephalography (EEG) was initiated. The patient was placed under general anesthesia. The left common femoral artery, known to be supplied by the false lumen, was surgically exposed and accessed via micropuncture technique. A 5F sheath was inserted, and an angled Glidewire (Terumo Interventional Systems, Somerset, NJ) and Berenstein catheter (Merit Medical, South Jordan, Utah) were passed into the false lumen of the abdominal aorta. Flush abdominal aortography demonstrated several large fenestrations (Fig 2, *A*). Under roadmap guidance, the proximal-most fenestration was crossed using the angled Glidewire and a Berenstein catheter, allowing access into the true lumen, which was then confirmed via intravascular ultrasound (IVUS) and angiography (Fig 2, *B* and *C*). Serial dilation of the fenestration up to 18F was performed. A 20F Gore DrySeal sheath (W.L. Gore & Associates, Flagstaff, Ariz) was then inserted across the fenestration, with the tip positioned in the true lumen (Fig 3). Through this, a Lunderquist wire was positioned in the ascending aorta, and a buddy wire was inserted to allow for positioning of a pigtail catheter, given the occluded contralateral iliofemoral system. Ascending and arch angiography was performed, which again demonstrated the dilated descending thoracic aorta and significant false lumen filling (Fig 4, *A*). A Navion stent graft 34 × 34 × 223 mm (Medtronic Inc., Santa Rosa, CA) was deployed just past the take-off of the debranched vessels in zone 0, followed by a 34 × 34 × 223 mm distal extension piece, which was landed in zone 5 at the level of the celiac trunk. Balloon angioplasty of all seal and overlap sites was performed. Completion angiography demonstrated immediate augmentation of the true lumen with patent cervical and visceral vessels, and no antegrade flow into the false lumen (Fig 4, *B*). Subsequently, the left brachial artery was accessed using micropuncture technique under ultrasound guidance. We then proceeded to coil the origin of the left subclavian artery, taking care to maintain patency to the vertebral artery.

**Fig 5:**
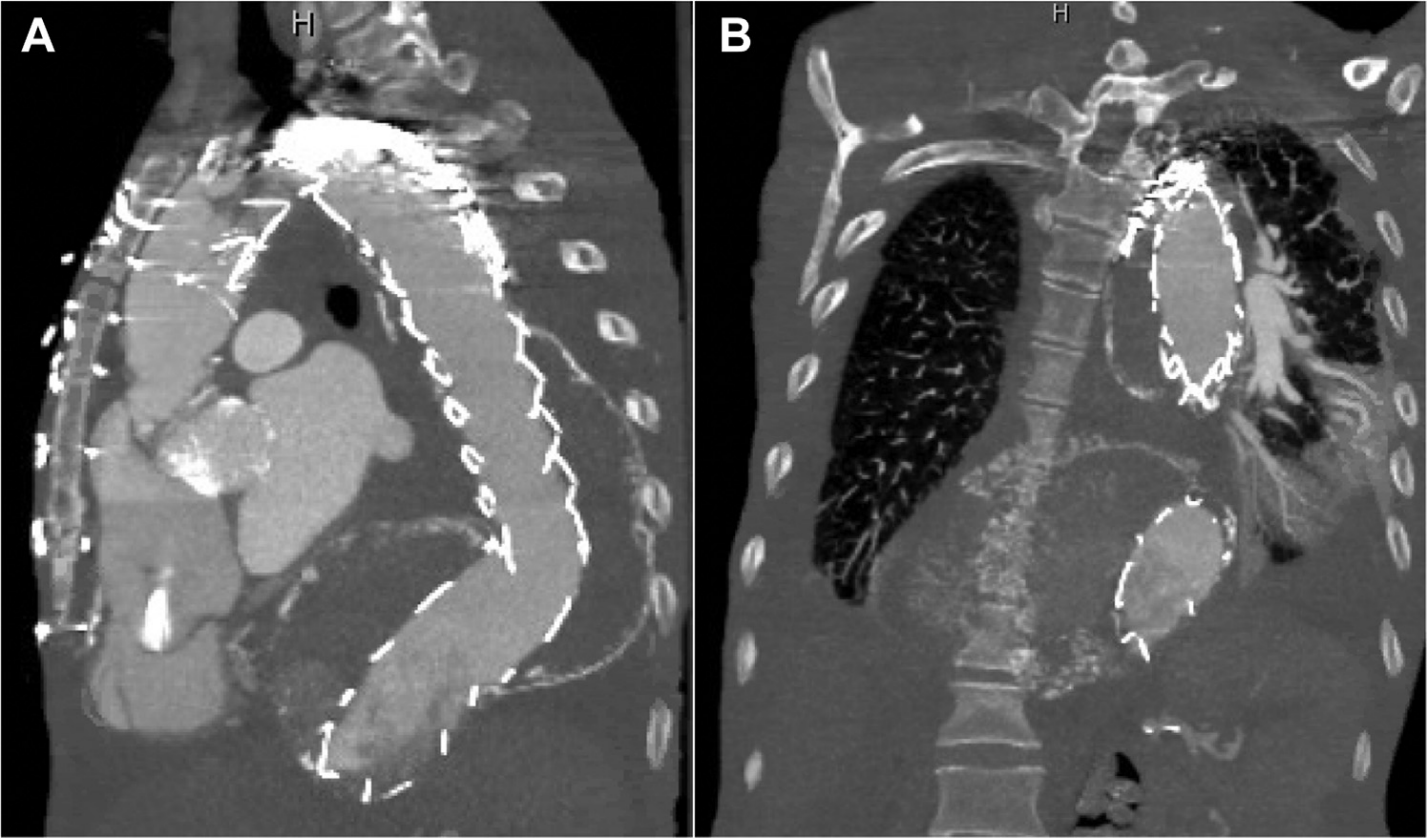
Sagittal **(A)** and coronal **(B)** views of the repair at 9 months. Note the significantly augmented true lumen at the level of the aortic hiatus and thrombosis of the false lumen in the descending thoracic aorta with a blush of contrast at the transition into the abdominal aorta, indicating residual retrograde flow from the perivisceral segment.

The patient's postoperative recovery was largely uncomplicated. She did require an extended stay because of several days of agitation. She was discharged on postoperative day 11 with no new deficits and mental status at baseline. Nine-month follow-up CTA demonstrated intact repair with positive aortic remodeling, augmentation of the true lumen distally, and near-complete thrombosis of the false lumen with residual retrograde filling from the visceral vessels (Fig 5).

## Discussion

TEVAR has become the preferred treatment method for thoracic aortic aneurysms.^1^, ^2^, ^3^, ^4^ In cases of chronic dissection, aneurysmal dilation is typically secondary to persistent flow in the false lumen. Thus, the goal of treatment is to augment the true lumen by preferentially redirecting flow, inducing false lumen thrombosis, and encouraging positive aortic remodeling. This is usually accomplished by coverage of the entry tear via endograft placement through bilateral groin access. Although alternative access methods have been described, the feasibility of TEVAR has traditionally been contingent on adequate femoral access.^6^^,^^7^

In the present case, the patient had an occluded right iliofemoral system, limiting us to unilateral access via the left iliofemoral system, which was supplied by the false lumen. Although techniques describing intentional endograft placement within the false lumen have been previously reported, initial access via the false lumen for endograft delivery has not been described.^8^ Furthermore, with our entry point in the false lumen, it was critical that we gained access to the true lumen for endograft placement. Reports have described manual creation of fenestrations via a variety of techniques; however, we emphasize here the use of preexisting fenestrations when feasible to minimize the possibility of complications.^9^, ^10^, ^11^ Serial dilations allowed us to safely pass a large-bore sheath for subsequent endograft delivery.

An alternative method of access that can be considered is construction of a right-sided iliac conduit because the right common iliac artery was patent and not dissected. This would allow for in-line access to the true lumen, an option to revascularize the hypogastric artery for additional spinal cord protection and allow reperfusion of the extremity. The patient was asymptomatic from the chronic right iliofemoral occlusion, already wheelchair-bound from the left lower extremity paralysis, mildly contracted on the right side, and she still had healing superficial ulcerations on her right hip and sacrum from prolonged immobility after her debranching surgery. Therefore, we decided the left groin was a more suitable access option.

In the context of complex thoracic aortic disease, many patients are not ideal candidates for open surgical repair because of concomitant comorbid conditions. Although the advent of TEVAR has allowed for a number of these patients to undergo treatment, there reamins a subset of patients with particularly challenging anatomy that may not initially appear amenable to TEVAR. Chronic dissections have proved to be particularly difficult, especially when branch vessel involvement is present. In the present report, we have added the use of natural fenestrations and false lumen femoral access as additional tools for the armamentarium in the treatment of patients with complex aortic dissections. Preoperative planning should include well-timed CTA to identify true and false lumen anatomy. A diagnostic angiogram, either as a separate session or at the time of operation, can confirm CTA findings and potentially identify additional aortic anatomic features not captured on CTA. The ability to customize treatment to each patient is critical when considering aortic intervention. In this case, we were able to take advantage of the existing features of the dissection to successfully deploy an endograft with early evidence of positive aortic remodeling.
